# The Incidence of Injury in Amateur Male Rugby Union: A Systematic Review and Meta-Analysis

**DOI:** 10.1007/s40279-017-0838-4

**Published:** 2018-01-03

**Authors:** Caithriona Yeomans, Ian C. Kenny, Roisin Cahalan, Giles D. Warrington, Andrew J. Harrison, Kevin Hayes, Mark Lyons, Mark J. Campbell, Thomas M. Comyns

**Affiliations:** 1 Department of Physical Education and Sport Sciences, University of Limerick, Limerick, Ireland; 2 Health Research Institute, University of Limerick, Limerick, Ireland; 3 Department of Clinical Therapies, University of Limerick, Limerick, Ireland; 4 Department of Mathematics and Statistics, University of Limerick, Limerick, Ireland; 5 Lero, The Irish Software Research Centre, University of Limerick, Limerick, Ireland

## Abstract

**Background:**

Rugby union is a physically demanding, full-contact team sport that has gained worldwide popularity. The incidence of injury in rugby union has been widely reported in the literature. While comprehensive injury surveillance and prevention programmes have been implemented within the professional game, there is a need for similar strategies in the amateur game. Despite recent increases in the volume of research in rugby, there is little consensus regarding the true incidence rate of match and training injuries in senior amateur male rugby union players.

**Objective:**

The aim of the current review was to systematically review the available evidence on the epidemiology of time-loss injuries in senior amateur male rugby union players and to subsequently conduct a meta-analysis of the findings.

**Methods:**

A comprehensive search of the PubMed, Scopus, SportDiscus and Google Scholar electronic databases was performed using the following keywords; (‘rugby’ OR ‘rugby union’) AND (‘amateur’ OR ‘community’) AND (‘injur*’ OR ‘pain*’). Six articles regarding the incidence of injury in senior amateur male rugby union players, in both matches and training, were retrieved and included in the meta-analysis to determine the overall incidence rate of match injury, with descriptive analyses also provided for other reported variables.

**Results:**

The overall incidence rate of match injuries within senior amateur rugby union players was 46.8/1000 player hours [95% confidence interval (CI) 34.4–59.2]. Contact events accounted for the majority of injuries, with the tackler more at risk than the player being tackled, and with respective incidence rates of 15.9/1000 player hours (95% CI 12.4–19.5) and 12.2/1000 player hours (95% CI 9.3–15.1).

**Conclusion:**

This meta-analysis found that the incidence rate of injury in amateur rugby union players was lower than that in professional players, but higher than the incidences reported in adolescent and youth rugby players. By understanding the true incidence and nature of injuries in rugby, injury prevention strategies can best be implemented. Future prevention strategies may best be aimed towards the tackle area, specifically to the tackler, in order to minimize injury risk.

## Key Points

**Table:** 

1. The overall incidence of match injury in amateur rugby union is 46.8/1000 player hours [95% confidence interval (CI) 34.4–59.2]. Forwards appear to have a higher incidence of injury than backs.
2. The incidence rate of injury in senior male amateur rugby union players appears to be lower than that in professional players, but higher than the incidences reported in adolescent and youth rugby players.
3. The incidence of injury is greater when tackling [15.9/1000 player hours (95% CI 12.4–19.5)] than when being tackled [12.2/1000 player hours (95% CI 9.3–15.1)].

## Introduction

Rugby has gained international popularity, becoming one of the most played and watched collision sports in the world, with approximately 8.5 million registered players in over 121 countries worldwide [1]. There are two major variants of rugby: rugby union and rugby league. Rugby union consists of two teams of 15 players competing to ground the ball over the opposition goal line by carrying, passing and kicking, while rugby league is played with teams of 13 players. Due to the differing laws and nature of contact events within these codes, as outlined by Freitag et al. [2] the incidence, mechanism and nature of injuries varies [3, 4]. In this review, rugby union (hereafter ‘rugby’) was the sole focus. Rugby has enjoyed increased popularity in recent years, with modified versions of the game emerging, such as Tag Rugby and Rugby Sevens. It was previously included in the Olympic Games in 1924, and returned in 2016 as an Olympic Sport, with the introduction of Rugby Sevens. Rugby is played in both amateur and professional settings, following the introduction of professionalism in 1995 [5]. It is an intensely physical game with numerous contact events and collisions, interspersed with periods of lower-intensity activity, such as walking and jogging [6]. The combination of high physical demands, alongside exposure to collisions and contacts, means the inherent risks of injury are substantial [7].

A meta-analysis evaluating the incidence of match injuries in senior professional male rugby players found an overall pooled incidence rate of match injuries of 81/1000 player hours [4]. In comparison, the incidence in adolescent and youth players has been found to be 26.7/1000 player hours [2]. While this is considered to be high in comparison to some sports, such as soccer and basketball, it is comparable with other collision sports, such as ice hockey, Australian Rules football and American football [8–11]. The incidence of injury in the amateur rugby game has been widely reported in various studies, ranging from 5.95/1000 player hours to 99.5/1000 player hours; however, inconsistencies in the methods of data collection and injury definitions used, make interstudy comparisons challenging [12, 13]. While it has been found that injury surveillance in amateur cohorts is more difficult than in professional cohorts owing to the lack of resources and the infrequent contact between medical professionals and amateur teams, consistent injury definitions and methods of data collection may provide much needed epidemiological information [14, 15]. The International Rugby Board (IRB), now called World Rugby, published a consensus statement on data collection and injury definitions in rugby in 2007, giving clear definitions for injury, recurrent injury, non-fatal catastrophic injury, classification of injuries, and training and match exposures [16]. These guidelines have led to an increase in the quality and consistency of research within rugby cohorts. In order to effectively minimize injury in sport, as outlined by the Translating Research into Injury Prevention Practice (TRIPP) Model, a full understanding of the incidence and etiology of injuries is required [17]. While many studies have aimed to establish the incidence of injury in rugby, the varying methods, injury definitions and length of follow-up make comparisons difficult [12, 18, 19]. By pooling data from several studies using comparable methodologies, overall estimates of injury incidence can be produced that more accurately reflect the injury incidence rate present among the amateur population [20].

## Objective

The aim of the current review was to systematically evaluate the available evidence on the epidemiology of injuries in senior amateur male rugby players and to conduct a meta-analysis of the findings. In order to accurately synthesize the incidence of injury, only prospective, epidemiological, observational studies and randomized controlled trials were included in this review.

## Methods

Guidelines for reporting Meta-analyses Of Observational Studies in Epidemiology (MOOSE guidelines) were adhered to in the format and reporting of this review [21]. The checklist contains specifications for reporting of meta-analyses of observational studies in epidemiology, including background, search strategy, methods, results, discussion and conclusion. A comprehensive search of the PubMed, SPORTDiscus, Scopus and Google scholar databases was conducted from January 1995 to October 2016. The following keywords were combined using Boolean operators to obtain relevant articles; (‘rugby’ OR ‘rugby union’) AND (‘amateur’ OR ‘community’) AND (‘injur*’ OR ‘pain*’). In addition, bibliographies of included studies and previous reviews were searched in order to identify other potentially eligible articles. Studies were limited to English-language articles from peer-reviewed journals. After removal of duplicates and reprints, titles and abstracts of articles were screened for suitability. A considerable number of citations were not relevant, as the keyword ‘rugby’ also encompassed articles pertaining to American football, rugby league, Australian football and/or soccer. Full-text articles were retrieved in order to determine inclusion or exclusion. In an attempt to reduce selection and recall bias, inclusion was limited to prospective cohort studies of injuries in rugby. Thus, review articles, retrospective studies, single or multiple case reports and case series were excluded. Although the definition of what constituted a reportable injury varied within the literature, no studies were eliminated on the basis of their operational definition at first.

Prospective cohort studies reporting the incidence of match injury, in 15-a-side senior amateur male rugby teams, over a minimum of one season, were included. Only studies reporting injuries as per the consensus guidelines were included in the meta-analysis. The definition of a ‘senior’ player was any player involved in adult amateur club rugby. This excluded colt teams (aged 17–19 years) and collegiate teams (aged 17–21 years) where it was not possible to extract the data pertaining specifically to adult amateur club players. However, studies involving a mix of rugby codes, age groups, or level of play were included provided separate data could be extracted for the desired cohort. Studies focusing on one particular injury type, without reporting an overall match incidence rate were excluded.

The full-text articles were retrieved and independently evaluated against the inclusion criteria by two reviewers (CY, RC). In the case of any disagreement over the suitability of a text, a third reviewer (IK) mediated.

General information pertaining to the number of participants involved, length of follow-up, and injury definition used was extracted from each of the included studies and compiled into a spreadsheet and summarized in Table 1. Only data required for this review were included from studies reporting the injury incidence rate for different age groups and levels of play. Where injury incidences were not reported per 1000 player hours, the following equation was used to calculate the incidence of injury (Eq. 1): [4]:

1 $${\text{Injury incidence}} = \frac{\text{no. of injuries}}{{{\text{no. of matches }} \times {\text{no. of players }} \times {\text{match duration}}}} \times 1000,$$

*Match duration, using the factor 1.33, based on standard 80-min game.

**Table 1:** Study characteristics, incidence of injuries and injury definition

References	Study duration	Injury definition	Level of play	Overall incidence rate (per 1000 player hours)
Bird et al. [30]	1 season^a^	All injury events that caused the player to seek medical attention or miss at least one scheduled game/team practice	Senior A Senior B	14.0 (12.0–16.2)^b^ 10.7 (7.5–14.7)^b^
Chalmers et al. [7]	1 season	Any event resulting in an injury requiring medical attention or causing a player to miss at least one scheduled game/team practice	Senior A Senior B/reserve Presidents/social Other	15.4^b^ 10.5^b^ 14.5^b^ 9.2^b^
Garraway et al. [5]	2 seasons	An injury sustained during a competitive match that prevented the player from training or playing rugby from the time of injury or the end of the match in which the injury was sustained	All registered amateur clubs in the Border Reivers district	22.6 (20.7–24.5)^c^ 14.8 (13.3–16.3)^d^
Roberts et al. [31]	3 seasons	Any injury incurred during a first-team training match resulting in an absence from participation in match play for 1 week or more from the day of injury	Group B (amateur) Group C (social)	16.6 (15.2–17.9) 14.2 (13.0–15.4)
Schneiders et al. [32]	1 season	Any physical event that occurred during a match that required a player to seek medical attention from a team doctor/physiotherapist and/or sports medic, or miss at least one scheduled game or team training	Premier grade (highest amateur level)	52 (42–65)
Swain et al. [33]	1 season	Any physical event that was caused by a transfer of energy that exceeded the body’s ability to maintain its structural and/or functional integrity, which was sustained by a player during a rugby match match/training, irrespective of the need for medical attention or time-loss from rugby activities	1st grade 2nd grade 3rd grade 4th grade	52.3 (43.7–62.2)

^a^Season = approximately 9 months

^b^Per 100 player games, as reported in the original article. Figures were then adjusted according to the previously mentioned equation in order to conduct meta-analysis per 1000 player hours

^c^Period prevalence figure 1997–1998 season used in meta-analysis for new and recurrent injuries

^d^Period prevalence figure1993–1994 season used in meta-analysis for new and recurrent injuries

Where the required incidences were not available or the methods of data collection required clarification, the corresponding authors of the original studies were contacted.

The reporting quality of the included articles were assessed using the Critical Appraisal Skills Programme (CASP) checklist for cohort studies. The checklist consists of 12 questions, with 2 initial screening questions and a further 10 questions exploring the results of the study and its validity and applicability to the local population [22].

The definition of what constituted an injury varied widely across the literature. Following the introduction of the consensus guidelines on data collection, injury recording and injury definition within rugby, the process has been streamlined, making cross-comparison easier [16]. The consensus guidelines define an injury as “Any physical complaint, which was caused by a transfer of energy that exceeded the body’s ability to maintain its structural and/or functional integrity, that was sustained by a player during a rugby match or rugby training, irrespective of the need for medical attention or time‐loss from rugby activities” ([16]; p. 193). It also differentiates between medical attention injuries (those that require a player to receive medical attention) and time-loss injuries (those that result in a player being unable to take a full part in future rugby training or match play) [16]. Only studies using the consensus guidelines injury definition, or a similar version of this definition, were included in the meta-analysis to ensure accuracy when comparing the incidences and nature of injury in rugby. Similar to a meta-analysis conducted in senior professional rugby players, only studies reporting injuries resulting in time-loss from matches or training were included in the meta-analysis [4].

The overall incidence was estimated using both fixed-effect and random-effect meta-analysis models using the R package, Meta [24, 25]. Inverse weighting was used in order to pool the incidences from the different studies, and a log transformation was then used to scale the estimated incidences in the respective models. The heterogeneity statistic *I*-squared and corresponding *p* value were also provided. Following the papers by Guddat et al. [26] and Higgins et al. [27], the results were gathered together and presented by means of a forest plot (Fig. 2).

In order to accurately discuss the differences in injury rates in the amateur game versus the professional game, data were analyzed as previously reported [4]. Comparisons of injury incidence data were made using a customized Microsoft Excel spreadsheet macro for combining effect statistics, whereby the incidence rate ratio (and its associated confidence limits) was assessed against a predetermined threshold of 0.91–1.10, indicated either a low or high risk, respectively [28, 29].

## Results

**Fig. 1:**
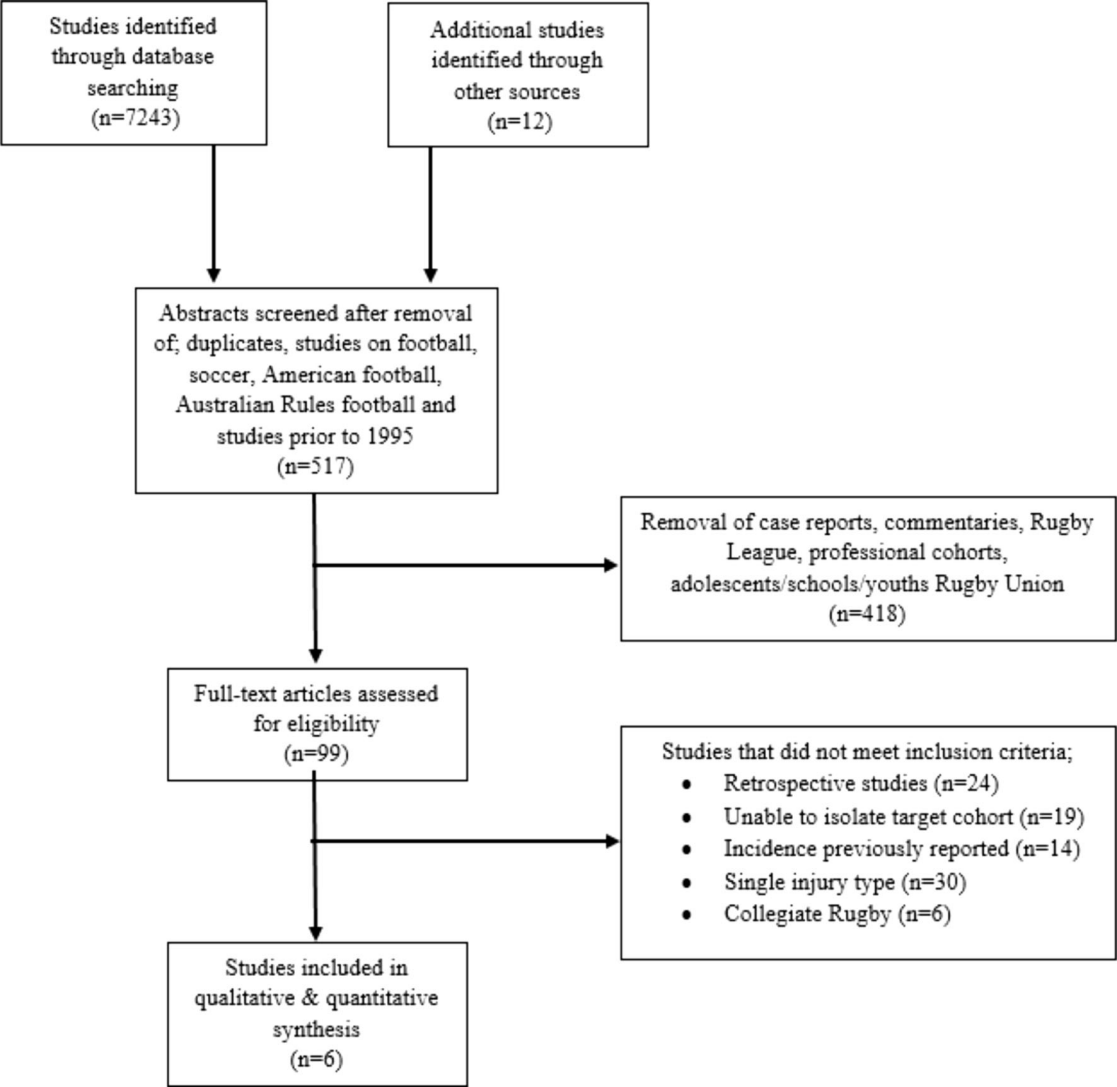
PRISMA [23] flowchart illustrating the inclusion and exclusion criteria used in the systematic review. *PRISMA* preferred reporting items for systematic reviews and meta-analyses

Overall, 7255 articles were identified using the search strategy outlined in Fig. 1. Following the removal of duplicates and articles discarded based on the full-text review, six articles were included for meta-analysis (Table 1).

### Critical Appraisal Skills Programme (CASP) Results

The results of the CASP assessment are shown in Table 2, excluding questions 7, 8 and 12, which pertain specifically to the incidence rates reported in each study. These reported incidence rates were extracted and are discussed in depth in the Results section.

**Table 2:** CASP checklist for cohort studies

References	CASP checklist										
	Q1	Q2	Q3	Q4	Q5(a)	Q5(b)	Q6(a)	Q6(b)	Q9	Q10	Q11
Bird et al. [30]	Yes	Yes	Yes	No	Yes	Yes	No	Yes	Yes	Yes	Yes
Chalmers et al. [7]	Yes	Yes	Yes	Yes	Yes	Yes	Yes	Yes	Yes	Yes	Yes
Garraway et al. [5]	Yes	Yes	Yes	Yes	Yes	Yes	Yes	Yes	Yes	Yes	Yes
Roberts et al. [34]	Yes	Yes	Yes	Yes	Yes	Yes	Yes	Yes	Yes	Yes	Yes
Schneiders et al. [32]	Yes	Yes	Yes	Yes	Yes	Yes	Yes	Yes	Yes	Yes	Yes
Swain et al. [33]	Yes	Yes	Yes	Yes	Yes	Yes	Yes	Yes	Yes	Yes	Yes

*CASP* critical appraisal skills programme

### Incidence of Injury

**Fig. 2:**
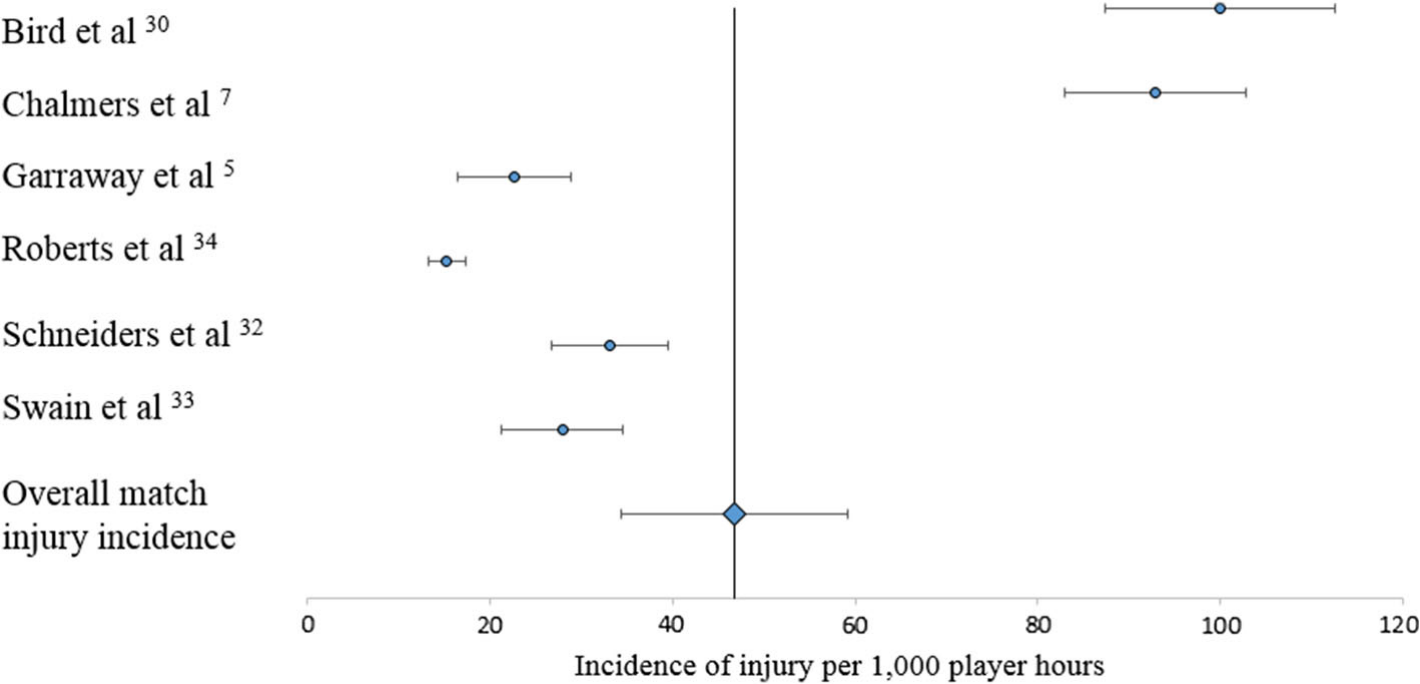
Incidence of match injuries (per 1000 player hours, with 95% confidence intervals). Roberts et al. [31] used an 8-day time-loss injury definition

The six studies included in the meta-analysis followed the consensus guidelines definition of an injury [5, 7, 30–33]. One study reported incidences for amateur and semi-professional players [31]. In order to accurately report the pooled incidence of injury for amateur players only, the figures reported for semi-professional players were excluded prior to conducting the meta-analysis. The six studies encompassed 2340 match injuries during 104,308 h of match exposure. The overall incidence of match injury in senior amateur rugby union was 46.8/1000 player hours [95% confidence interval (CI) 34.4–59.2] (Fig. 2). Only one study reported the incidence rate of training injuries, therefore training injuries were not included in the meta-analysis [30].

Level of play was reported in four of the studies, with incidence rates separated according to specific levels [7, 30, 31, 33]. Given the different cohorts used in each study, it was not possible to compare the levels of play between the studies and therefore meta-analysis for this variable was not conducted. Bird et al. [30] reported separate incidence rates for Senior A (‘premier grade’ or highest-level amateur) and Senior B (second highest-level amateur) players, with a higher incidence of match injury found in the Senior A players. Similarly, Roberts et al. [31] reported a higher incidence of injury in group B (amateur) than group C (recreational and social). While Senior A players had the highest incidence of injury in one study, it was followed by the Presidential/Social cohort, with the lowest incidence observed in the Senior B/Reserve players [7]. Conversely, Swain et al. [33] separated the incidence of injury into Grades 1–4, with the 3rd Grade reporting the highest incidence, followed by the 4th grade, with the highest level of play (1st Grade) reporting the lowest incidence of injury.

### Injury Severity

Injury severity was reported in three of the included studies, in adherence with the consensus guidelines [31–33]. Injury severity, as per the statement, ranges from slight (0–1 day’s absence), minimal (2–3 days’ absence), mild (4–7 days’ absence), moderate (8–28 days’ absence) and severe (over 28 days’ absence) [16]. Pooled incidence rates for moderate and severe injuries were 7.6/1000 player hours (95% CI 7.3–7.9) and 3.7/1000 player hours (95% CI 3.1–4.3), respectively. Pooled incidence rates for slight, minimal and mild injuries were not possible to determine due to insufficient data [31, 32].

### Mechanism of Injury

The contact event accounted for the highest proportion of injury across the six studies, ranging from 48 to 80% [5, 31]. Two of the studies described the phase of play, during which an injury occurred, and provided the relevant information suitable for meta-analytic review [32, 33]. The tackle phase accounted for the majority of injuries reported. It was found that the tackler had an increased risk of injury compared with the player being tackled [15.9/1000 player hours (95% CI 12.4–19.5) and 12.2/1000 player hours (95% CI 9.3–15.1), respectively]. Using a customized Microsoft Excel spreadsheet macro, it was found that tackling carried an 84.2% true chance of injury risk, which was higher than the risk to the ball carrier [28]. The tackle event was the most prominent injury event, followed by the ruck [7.6/1000 player hours (95% CI 4.4–10.7)].

### Position

Three studies reported the incidence rate with respect to player position; however, meta-analysis for specific positions was not possible as the studies used the terms ‘forwards’ and ‘backs’ to describe the player positions [31–33]. ‘Forwards’ refers to props (numbers 1 and 3), the hooker (number 2), locks (numbers 4 and 5), flankers (numbers 6 and 7), and the ‘number eight’, while ‘backs’ refers to the fullback (number 15), wingers (numbers 11 and 14), centres (numbers 12 and 13) and the halfbacks (numbers 9 and 10). It was found that the forwards, with an incidence rate of 22.8/1000 player hours (95% CI 17.5–27.1), were more at risk than the backs, with an incidence rate of 18.1/1000 player hours (95% CI 13.7–22.5). Using the customized Microsoft Excel spreadsheet macro to compare the mean effects, it was found that the forwards had a 78.9% true chance of injury risk, which was higher than the risk to backs [28].

### Injury Type and Nature

**Fig. 3:**
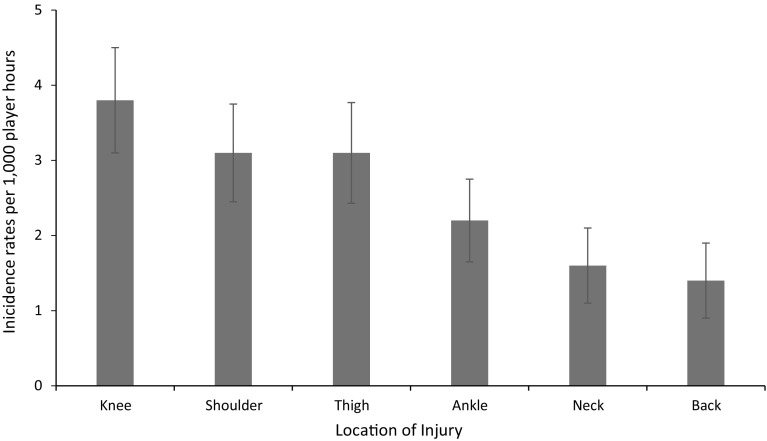
Incidence of injuries (with 95% confidence intervals) by location of injury

Four studies reported either the bodily location of injury and/or the nature of injury [5, 31–33], whereas three studies addressed the specific location of injury and were included for meta-analysis [5, 32, 33]. The remaining study grouped the incidence rates for injury location into either head, trunk, upper limb or lower limb; it was not possible to isolate the specific locations for the amateur cohorts and therefore the study was not included in the meta-analysis [31]. The knee was the most commonly injured joint, with a pooled incidence rate of 3.8/1000 player hours (95% CI 3.1–4.5). This was followed by the shoulder and thigh, both with an incidence rate of 3.1/1000 player hours (95% CI 2.4–3.7). Figure 3 shows the pooled incidence rates for the injury locations reported across the three studies. The study that could not be included in the meta-analysis for injury location reported a higher incidence of lower limb injuries compared with upper limb, head or trunk injuries [31].

**Fig. 4:**
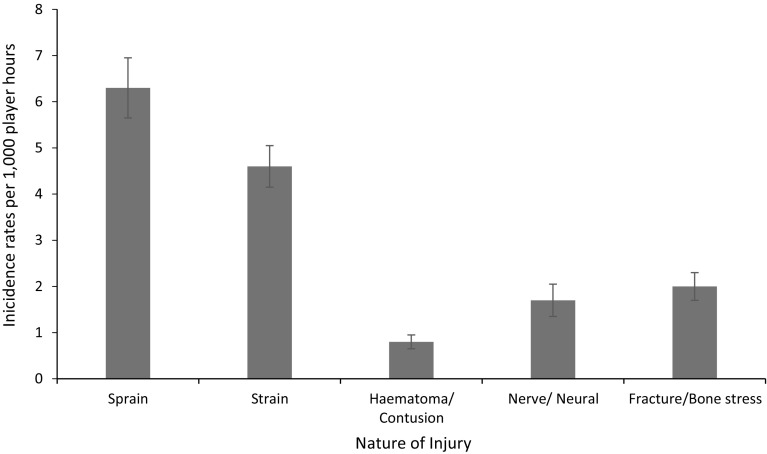
Incidence of injuries (with 95% confidence intervals) by injury type

Three studies reported the nature of injury and were included in the meta-analysis [31–33]. Sprains had the highest incidence of injury, followed by strains, with respective incidence rates of 6.3/1000 player hours (95% CI 5.6–6.9) and 4.6/1000 player hours (95% CI 4.2–5.1). Figure 4 shows the pooled incidence rates for the nature of injury reported across the three studies.

## Discussion

The results of this meta-analysis confirm that the match injury incidence rate of 46.8/1000 player hours in amateur rugby is low in comparison with professional cohorts, with a pooled incidence rate of 81/1000 player hours, but higher than the incidence rate of 26.7/1000 player hours seen in youth and adolescent teams [2, 4]. The results are similar to those previously reported in youth academy players [47/1000 player hours (95% CI 39–57)] and Under-17 amateur players [49.3/1000 player hours (95% CI 25.8–72.7)] [35, 36]. This mirrors the results reported in both amateur and professional soccer and rugby league, with a number of reasons hypothesized to account for this [15, 37].

### Data Collection

Within the professional set-up, reporting of injuries has been standardized, with qualified medical professionals present at all matches and training sessions [38]. In comparison, the current review has identified that injury surveillance varied widely across the amateur game. Two studies relied on the player disclosing an injury (via weekly telephone calls, interviews or questionnaires), with reported compliance rates of 92.7 and 88%, respectively [7, 30]. These studies had high incidence rates relative to our pooled estimate, and while player recall may be an accurate method of collating injury rates [39], the accuracy of these diagnoses may be questioned as no follow-up assessments with trained healthcare professionals were arranged. Chalmers et al. [7] recorded each injury event as a new injury, without ascertaining if the injury was a recurrence of a previous injury. It was also observed that players who continued playing while injured had a 46% higher risk of in-season injury [7]. This may account for the high incidence rate reported as players who did not believe their injury to be serious or wished to continue playing may have reported an injury exacerbation as a new injury episode if they were not fully rehabilitated [40]. Bird et al. [30] also reported a high injury incidence rate relative to our pooled estimate, and noted that many players may continue to play while injured. If players are not fully rehabilitated prior to returning to play, they may be at greater risk of re-injury and therefore the resultant injury incidence rates may have been higher [41]. Garraway et al. [5] arranged for follow-up physiotherapy appointments to ensure accuracy in injury reporting and diagnosis; however, injury rates may have been underreported due to injuries being missed, not diagnosed, or indeed resolved by the time an assessment was conducted [17, 42]. This relative effect has also been found in amateur soccer, where the infrequent contact between medical professionals and amateur teams often resulted in minor injuries being missed or not diagnosed, resulting in a lower incidence of injury in comparison with professional soccer teams [15]. One study used a ‘trained recorder’ associated with the research project to record injuries; however, it was unclear whether this was a trained medical professional, therefore the accuracy of injury diagnosis may be subject to scrutiny [33]. The remaining studies relied on injury reports from the associated team physiotherapist, doctor, sports trainer, athletic trainer or coach [31, 32]. Hagglund et al. [43] highlighted concerns regarding the consistency of injury data collection from multiple observers, in the development of the Union of European Football Associations (UEFA) Football Safety Project. While the accuracy of injury recognition and diagnosis is reliant on the skill of the observer, regardless of level of play, care must be taken when interpreting results, particularly in the amateur game where medical support may often be less consistent secondary to economic restraints of amateur teams [15]. Adherence to available consensus guidelines, and adequate training of the injury recorders, may result in more accurate data.

### Level of Play

The intensity of training sessions and matches increases with competition and therefore highly skilled players may experience a greater risk of injury than less-skilled players [44]. While meta-analysis on the level of play was not possible, three studies reporting differences in the injury incidence rate according to level of play observed an increased incidence with teams at a higher level [7, 30, 31]. Only one study reported the lowest incidence of injury in the team at the highest level of play [33]. Within the professional rugby cohorts, a higher level of play was associated with a higher incidence of match injury, with proposed explanations including increased levels of competitiveness and the increased size and strength of players [4]. Ekstrand et al. [45] reported that the increased intensity at the professional level in soccer may contribute to the higher number of injuries found. Similarly, Hopper et al. [46] reported that athletes with the highest skill level were more likely to incur injury than less-skilled athletes due to the higher level of competitiveness, combined with a longer season. Palmer-Green et al. [35] reported a 34% higher injury incidence in the professional youth academy cohort (high level) than the amateur school cohort (lower level), which may have been due to the higher collision forces and increased number of contact events in the academy cohort. Another explanation for this is the increased size and strength of professional players [47]. Players at a higher level may be fitter, faster and stronger, therefore increasing the collision forces within contact events throughout the game, resulting in a higher number of injuries [48]. Gabbett [44] reported that the increased physiological capacity of semi-professional rugby league players may account for the higher incidence of injury found compared with the amateur cohort as this may result in a higher playing intensity. The results of this meta-analysis found a higher incidence of injury in senior amateur rugby players than in youth or adolescent cohorts, where a pooled incidence rate of 26.7/1000 player hours has been previously reported [2, 49]. This is likely due to the increased size and strength of more senior players, resulting in higher forces in contact events [50]. Garraway et al. [5] observed an increased incidence of injury within the amateur cohort from the 1993–1994 season to the 1997–1998 season. Similar methods were followed for the same cohort in the 1993–1994 pre-professionalism season, for ease of comparison. While it is possible that the increase in incidence rate was due in part to improvements in injury recognition and reporting, the advent of professionalism within the sport may have also contributed [51].

### Injury Severity

The data regarding injury severity was found to be inconsistent, likely due to poor follow-up, inadequate injury reporting, and the inconsistent level of rehabilitative care available [43]. The incidence rate of severe injuries within the professional cohort has been found to be 15.1/1000 player hours (95% CI 10.5–21.7) [38], whereas severe injury incidence rates as low as 1.16/1000 player hours have been reported in younger cohorts [8]. The incidence rate of severe injuries, with a rate of 3.7/1000 player hours, in this study was found to be lower than that in the professional cohort, but higher than the adolescent players. One possible explanation for the low rate of moderate and severe injuries is that injuries occurring late in the season may not be followed up as rigorously by the player and/or team medical or coaching staff in an amateur setting [42]. While follow-up physiotherapy assessments were arranged in one study, this was just for accuracy of injury recognition and diagnosis, with no long-term follow-up regarding injury severity recorded [30]. One barrier to injury surveillance within an amateur set-up is the availability of qualified medical professionals to assess, diagnose and rehabilitate injury, and this is evident in the discrepancies in follow-up periods following injury and lack of injury severity data [42, 43]. The challenges of rigorous follow-up in the amateur game has been acknowledged and future research into injury surveillance and prevention in the amateur game needs to account for this [52]. New injuries occurred more often than recurrent injuries, which is in line with observations reported in the professional game [4]. While recurrent injuries account for a small proportion of injuries observed, it is important to recall the lack of follow-up in many of the amateur studies. Most data collection took place during match events and therefore overuse or recurrent injuries may have been misdiagnosed as a new acute injury due to a lack of baseline information.

### Mechanism of Injury

In recent times, injury prevention programmes in rugby have addressed the scrum as an area of concern with regard to incidence of injury [53–55]. With effective implementation, the incidence of scrum-related catastrophic injuries has decreased [56, 57]. Despite this decrease, the results of this meta-analysis found that forwards had a higher incidence rate of injury compared with backs; however, only three of the six included studies reported injury incidence rates in relation to position and were included in the meta-analysis. Appropriate statistical adjustments for exposure hours and player numbers for the three relevant studies may explain the higher incidence of injury observed in the backs, and also why the rates for position-related injuries appear as approximately half of the overall incidence. Coinciding with the decrease in scrum-related injuries, an increase in the incidence rate of catastrophic injuries resulting from open play was reported [57]. The results of this meta-analysis showed that the tackle event, specifically injuries to the tackler, accounted for the highest incidence of injury [15.9/1000 player hours (95% CI 12.4–19)], with the ball carrier at a lower risk [12.2/1000 player hours (95% CI 9.3–15)]. Bird et al. [30] and Garraway et al. [5] reported that the tackle phase of play accounted for the majority of injuries, i.e. 40 and 48% respectively, while Roberts et al. [31] reported 80% of all injuries occurred due to contact events. Similarly, the tackle event resulted in more injuries in the professional rugby cohort, however it was found that the ball carrier was at more risk of injury, with a pooled incidence rate of 29/1000 player hours, than the tackler (19/1000 player hours) [4]. The results of this meta-analysis found lower incidence rates for the tackle event compared with adolescent cohorts, likely due to the lower number of studies included in this meta-analysis. The adolescent cohort, similar to the professional cohort, reported a higher incidence of injury to the tackler than the ball carrier (18.5/1000 player hours and 16.5/1000 player hours respectively) [2]. The increased size and strength of more senior players may result in higher forces in contact events compared with youth or adolescent players, and future research should investigate the lower incidence rates for the tackle event in senior amateur players compared with adolescents [2, 50]. While the force of the collision may be greater at higher levels of play, there may also be a greater number of contact events during match play [58]. King et al. [59] reported that increased contact events at a higher playing intensity results in a higher incidence of injury in rugby league. It has also been reported that tackle events can vary depending on different levels of play, with elite or professional players engaged in more active shoulder tackles than younger players [60]. The differences in style of play and nature of tackle events may also have an effect on the overall match injury incidence rates reported in both professional, amateur and adolescent players [2, 4].

## Limitations

One limitation of this study was the low number of studies included for meta-analytic review. The varying injury definitions, duration of data collection and methodological differences resulted in only six studies being suitable for the meta-analysis. Despite the 2007 IRB consensus statement regarding data collection, and the subsequent improvements in the methodological quality of published studies, it is difficult to ensure consistency in reporting and data collection practices across studies and teams. Injury incidence rates have been measured and reported as per 1000 player hours, per 1000 athlete exposures and per 100 games or training sessions. The varying methods of reporting injury can often lead to differing conclusions being drawn, and thus results of studies should be interpreted with caution. Furthermore, one study only reported on time-loss injuries resulting in a minimum of 1 week’s absence from play, whereas the remaining studies defined a ‘time-loss injury’ as any injury that resulted in a missed match or training session. This may result in the pooled incidence rate found in the current paper to be lower than expected. Future studies should adhere to the aforementioned consensus statement in order to accurately report time-loss and medical attention injuries in rugby. Some studies combine match and training injuries, however this can be misleading when interpreting incidence rates, given the differences in hours of exposure and levels of competitiveness. Therefore, match and training injury incidences were extracted and only the incidence of match injury was analyzed to address this limitation.

## Conclusion and Future Directions

The primary objective of this review was to synthesize and analyze the incidence, severity and nature of injuries in senior amateur rugby union. The overall incidence of match injury in senior amateur rugby was 46.8/1000 player hours (95% CI 34.4–59.2). The incidence of injury in senior amateur rugby is lower than that reported in the professional game (81/1000 player hours), but higher than that reported in youth and adolescent cohorts (26.7/1000 player hours) [2, 4]. The tackle event accounted for the highest risk of injury. Epidemiological analysis plays a pivotal role in injury prevention, providing data required for development, application and assessment of injury causation and injury prevention models [52]. Understanding the nature, mechanism and surrounding events is the vital first step for injury prevention in rugby. While injury surveillance and player monitoring has been widely adopted within the professional game, attention must now be given to the amateur game [61–63]. By implementing a comprehensive injury surveillance programme, adhering to the IRB consensus guidelines, successful injury prevention strategies may be monitored at any level of the game. Future studies should adhere to the IRB consensus guidelines to allow for ease of comparison across studies. Collating this level of data will help clarify the true risk of injury and will streamline decisions as to where injury prevention strategies are best focused in order to reduce the injury risk and subsequent injury burden in amateur rugby.
